# Combined Enzymatic and Mechanical Cell Disruption and Lipid Extraction of Green Alga *Neochloris oleoabundans*

**DOI:** 10.3390/ijms16047707

**Published:** 2015-04-07

**Authors:** Dongqin Wang, Yanqun Li, Xueqiong Hu, Weimin Su, Min Zhong

**Affiliations:** 1 College of Food Science and Technology, Guangdong Ocean University, Zhanjiang 524088, China; E-Mails: wdqfighting@163.com (D.W.); hxwz247@163.com (X.H.); zhongminer_zj@126.com (M.Z.); 2 Guangdong Provincial Key Laboratory of Aquatic Product Processing and Safety, Zhanjiang 524088, China; E-Mail: hdsuwm@163.com

**Keywords:** microalga, cell disruption, ultrasonication, high pressure homogenization, enzymatic lyses

## Abstract

Microalgal biodiesel is one of the most promising renewable fuels. The wet technique for lipids extraction has advantages over the dry method, such as energy-saving and shorter procedure. The cell disruption is a key factor in wet oil extraction to facilitate the intracellular oil release. Ultrasonication, high-pressure homogenization, enzymatic hydrolysis and the combination of enzymatic hydrolysis with high-pressure homogenization and ultrasonication were employed in this study to disrupt the cells of the microalga *Neochloris oleoabundans*. The cell disruption degree was investigated. The cell morphology before and after disruption was assessed with scanning and transmission electron microscopy. The energy requirements and the operation cost for wet cell disruption were also estimated. The highest disruption degree, up to 95.41%, assessed by accounting method was achieved by the combination of enzymatic hydrolysis and high-pressure homogenization. A lipid recovery of 92.6% was also obtained by the combined process. The combined process was found to be more efficient and economical compared with the individual process.

## 1. Introduction

Rapid-growing global consumption of fossil fuel has caused not only crude oil shortage but also global warming and environment contamination. Microalgae can capture parts of the solar energy and mitigate atmosphere CO_2_. They also have much higher capability to produce biomass than terrestrial plants based on their higher rate-of-growth, as a kind of photosynthesis microorganism, and they can yield more oil under certain conditions than soybean and oil palm [1]. Some species of microalgae can accumulate lipid up to 50% *w*/*w* of their dry biomass and an oil yield of 0.13 g·L^−1^ cultivation broth per day [2]. In recent years, microalgae have attracted considerable attention due to their oil producing ability for biodiesel. However, the commercialization of this renewable, environmentally-friendly biofuel is limited by its high production cost. In order to increase the competitiveness of microalgal biodiesel, various researches have focused on its key processing stages, such as algal strain improvement [3], cell cultivation optimization [4,5,6,7,8], biomass harvest [9,10], oil extraction [11,12,13,14] and oil transesterification [15]. Algal oil extraction techniques can be briefly classified as dry and wet processing. In the dry processing, oil is extracted after algal biomass has been dried, and the drying exerts as much as 75% of the overall cost in the algae processing [16]. In the wet processing, the direct extraction of oil from the wet fresh algal cells avoids the major cost from the drying of biomass. However, algal oil is enclosed in cell by tough cell wall and membrane. Thus, algal cell disruption is a key factor to liberate oil out of cell to facilitate the extraction.

The cell wall in most of the green microalgae typically consists of polysaccharides (such as cellulose, pectin and/or algaenan) and proteins (such as glycoproteins) [17]. The tensile strength of the cell wall can be up to ~9.5 MPa, which is about the same as bacteria or yeast, but three times higher than that of high plant cells such as from carrot, *Daucus carota* [18]. Many protocols have been used for microalgal cell disruption, including mechanical methods [19,20], chemical and heating treatments [21,22], microwave treatments [23] and enzymatic hydrolyzation [24]. Mechanical disruption is generally preferred as it generally avoids further chemical contamination of the algal preparation and preserves most of the functionality of the cellular materials. Ultrasonication, high pressure homogenization and bead milling are three of the most widely used mechanical methods [19,20,24]. Mechanical treatments can usually give some kind of strong force, such as shear stress, acting on the cell wall, so the cell wall usually can be torn directly into pieces. In the reported results [19,20,23,25], the cell disruption efficiencies achieved by mechanical methods are usually relatively high compared with other methods.

Ultrasonication works by the oscillations of the probe to create unsteady cavitations, which implode with extremely localized shock waves, so that large forces are provided to disrupt cells. The cavitation also produces micro-scale eddies which can induce stress acting on algal cells. Ultrasonication has been intensively used for microalga cell disruption [26,27,28,29]. However, reports showed that ultrasonication has not given very high disruption efficiency and it costs much energy [30].

Homogenization is widely used in industry. Since homogenizer can give strong shear stress and impact on the cells, it usually gives a high efficiency of cell disruption. In homogenization processing, pressure is considered a key factor. The pressure required depends on the different cell wall structures of different microorganism species. A recent study [31] reported that the pressure required to achieve rupture of 50% of the cells per pass was 17, 107, 138, and 200 MPa for *Tetraselmis* sp.; *Chlorella* sp.; *S. cerevisiae*; and *Nannochloropsis* sp., respectively. However, high pressure homogenization also requires high energy; for example, a high pressure homogenization of five consecutive passes at 52–86 MPa was used in a research report [19].

Enzymatic process, as a biochemical process, requires much lower energy than mechanical processes. Because the constitution of green microalga cell wall and the contained cellulose are similar to most plant cells [32], it is expected that the algal cell wall can be weakened and loosened by degradation of the protein, cellulose and/or pectin of the wall. Researchers have reported many results of enzymatic degradation [10,24]; however, the enzymatic process has not achieved very high cell disruption degree in the reports. Most of reported studies have used enzymes separately, and have also had limited lipid extraction; not more than 72% of lipid recovery has been achieved [33,34]. For improving the enzymatic disruption of algal cells, synergy of different kinds of enzymes might be applied.

*Neochloris oleoabundans* is a freshwater green microalga species, belonging to the *Chlorococcaceae* family [35]. It can accumulate up to 50% of lipid in its dry biomass under photoautotrophic conditions [2], and 80% of its total lipids are triglycerides and most of the fatty acids are saturated fatty acid in the range of 16–20 carbons [36], so it is ideal for biodiesel production. The cell size of *N. oleoabundans* ranges from 2 to 6 μm and the cell behaves like high strength bacteria, so that the cell disruption is hard work, like other microalgal cell disruption.

Most microalgal lipid extraction studies are based on sole technique, and the disruption degrees and lipid recoveries obtained are not acceptably high, but the energy consumptions for the lipid extraction are still too much [37]. Considering the cell wall skeleton of *N. oleoabundans* is dominantly built from cellulose and embedded with proteins in the cell wall, an enzymatic hydrolyzation with cellulase and proteases are combined with homogenization or ultrasonication to disrupt microalgal cells, and their efficiencies of cell disruption and lipid extraction are evaluated to find an efficient and economical microalgae oil extraction process.

## 2. Results and Discussion

### 2.1. Cell Disruption by Ultrasonication

**Figure 1:**
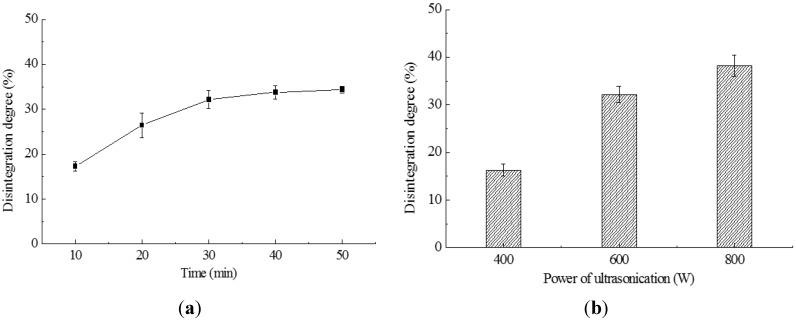
Effects of ultrasonic treatments on the disruption degree. (**a**) The ultrasonication power was 600 W; (**b**) the ultrasonic treating time was 30 min.

The results of cell disruption by ultrasonication are shown in Figure 1. The disruption degree increased with the treating time, as shown in Figure 1a, but the increase was little after 30 min. According to this result, the operation time of ultrasonication in the later application could be selected as 30 min. Figure 1b shows that the disruption degree increases with the ultrasonic power, doubling when the ultrasonic power rose from 400 to 600 W. However, the increase of disruption degree was only 6% when the power rose from 600 to 800 W. Therefore, the ultrasonication power could be selected at 600 or 800 W, and a higher power than 800 W can produce only little disruption.

### 2.2. Cell Disruption by High Pressure Homogenization

**Figure 2:**
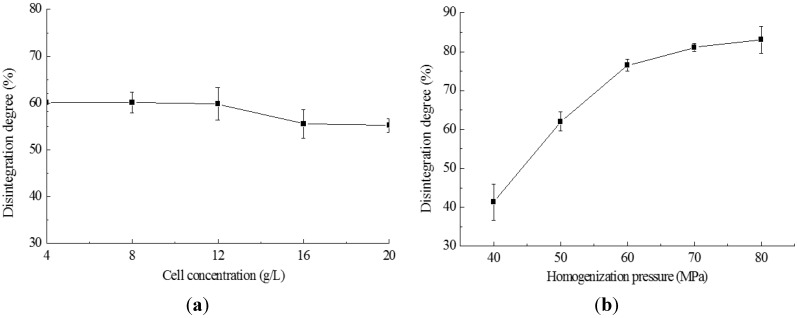
Effect of homogenization on the degree of cell disintegration. (**a**) The homogenization pressure was 50 MPa; (**b**) the suspension was at 20 g/L and homogenized for two passes.

In homogenization processing, the effects of alga biomass concentration on the disruption degree are shown in Figure 2a, indicating that biomass concentrations from 4 to 20 g/L dry cell weight did not influence the disruption degree significantly (*p* < 0.05). These results show that the biomass concentration did not require special adjustment during processing. The homogenization pressures of 40–80 MPa tested here showed that the homogenization pressure affected the cell disruption significantly (*p* > 0.05) (Figure 2b). The largest change (35.2%) of the disruption degree occurred when the pressure was increased from 40 to 60 MPa, and 4.6% of the disruption degree increase was obtained when the pressure increased from 60 to 70 MPa, but only 2.0% of disruption degree increase happened when the pressure increased from 70 to 80 MPa. In view of this result, the operation pressure of homogenization could be selected to be 60 MPa.

### 2.3. Cell Disruption by Enzymatic Lyses

**Figure 3:**
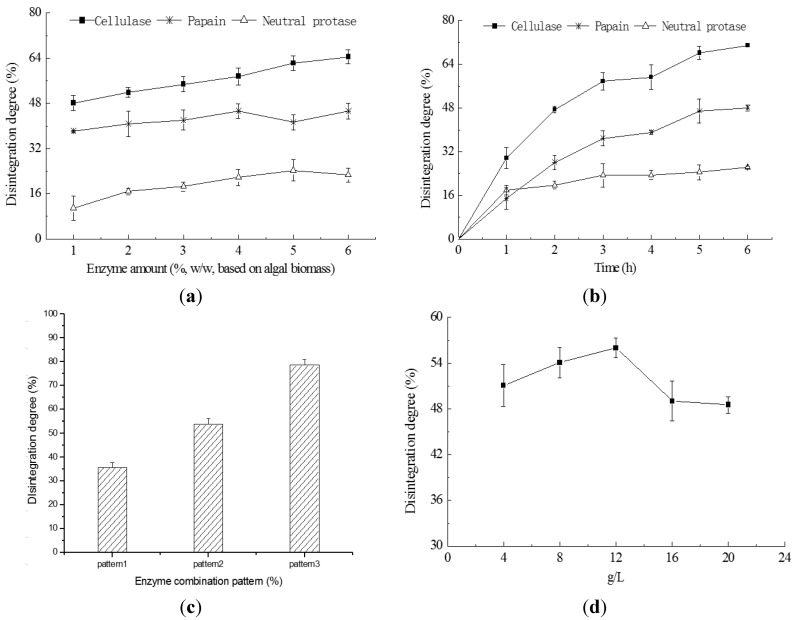
Effects of enzymatic treatments on the degree of cell disintegration. (**a**) The treatments performed at the optimum temperatures of the enzymes (cellulase 50 °C, papain 40 °C, respectively), with cell concentration 4 g/L and pH 6.5; (**b**) The quantity of cellulase was 5% (*w*/*w*), papain 3% (*w*/*w*) and neutral protease 3% (*w*/*w*), with cell concentration 4 g/L; (**c**) In pattern 1, 2% (*w*/*w*) papain and 3% (*w*/*w*) cellulase were combined; in pattern 2, 3% (*w*/*w*) cellulase acted for 3 h and 2% (*w*/*w*) papain followed, acting for 2 h; in pattern 3, the lyses was performed by 2% (*w*/*w*) papain for 2 h and then by 3% (*w*/*w*) cellulase for 3 h, with the cell concentration 20 g/L; (**d**) Treated by 2% (*w*/*w*) papain for 2 h and by 3% (*w*/*w*) cellulase for 3 h.

The effects of enzyme amount on the cell disruption degree are presented in Figure 3a. The ratio of enzyme mass to alga biomass was 1% to 6% in the experiments. Cellulase achieved the highest disruption degrees from 48.2% to 64.4%, while the lower disruption degree was obtained by papain (from 38.1% to 45.2%) and the neutral protease performed the weakest (from 10.9% to 24.3%). The effects of treating time on the cell disruption are shown in Figure 3b. The disruption degree rose with the increase of the treating time, and most of the disruption was obtained in the first 3 h of the treating time.

In the processes of enzyme combinations, there were significant differences (*p* < 0.01) in disruption degree between the combination patterns (Figure 3c). Pattern 3 possessed a much higher disruption degree than the other two patterns.

Cell concentrations of the algal suspension were also tested, but failed to demonstrate significant difference (*p* < 0.05) in the disruption degree within the tested concentration range (Figure 3d).

### 2.4. Cell Disruption by Combined Processes

**Figure 4:**
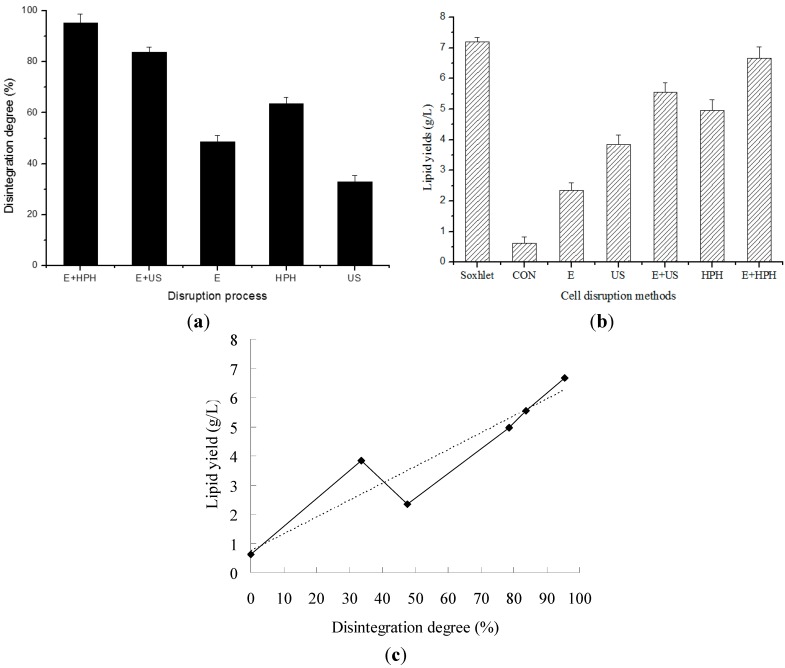
The cell disruption degree and lipid extraction yield in different processes. One-liter algal cell suspension at 20 g/L of biomass concentration was firstly treated by disruption and then the lipid was extracted. The disruption conditions were as follows: “CON”, control, the cells were not disrupted; “US”, ultrasonicated at 600 W for 30 min; “HPH”, homogenized one pass at 60 MPa; “E”, treated by 1% (*w*/*w*) papain for 2 h followed by 1% (*w*/*w*) cellulase for 3 h; “E + HPH”: Treated by 1% (*w*/*w*) papain for 2 h followed by 1% (*w*/*w*) cellulase for 3 h and then homogenized one pass at 60 MPa; “E + US”: 1% (*w*/*w*) papain for 2 h followed by 1% (*w*/*w*) cellulase for 3 h, and then ultrasonically treated at 600 W for 30 min. (**a**) The disintegration degree of different disruption processes; (**b**) lipid yields of different disruption processes and (**c**) the relationship of lipid yield with integration degree.

The results of combinations of enzymatic lyses with high-pressure homogenization and ultrasonication are shown in Figure 4a. The combination processes were also compared to mechanical and enzymatic sole processes. In the combined processes, the quantity of enzymes used was reduced compared to the enzymatic process described above (Figure 3), and the mechanical operation strength was also lower but the disruption degree of the combined processes was higher than that of any other sole process. Figure 4b shows that mechanical disruption processes supplied more lipid production than enzymatic disruption process, and that homogenizing process contributes higher lipid production than ultrasonic processes. Moreover, the lipid yield of combined processes was much higher than that of the sole processes.

The lipid content of the harvested microalgal biomass was 7.2 g lipid per L of suspension (mass concentration was 20 g/L) as measured with Soxhlet method and the equivalent lipid content of cell biomass was 36% of dry cell weight. The lipid recovery achieved by direct extraction from undisrupted cell was very low (Figure 4b), which was about 10%. In the combination process, a reduced quantity of enzymes compared to sole enzymatic process (papain: 2% to 1%; cellulase: 3% to 1%) was co-applied with less mechanical treatment (high-pressure homogenization, one pass at 60 MPa; ultrasonication: 800 to 600 W), as a result a significant increase of lipid yield (*p* < 0.05) was achieved. Based on the lipid yield of Soxhlet method, the highest lipid recovery of enzyme-homogenization combined process was 92.6%.

When the lipid yields obtained from each different extraction process were plotted with the corresponding disintegration degree, a relationship curve could be obtained (Figure 4c). A raising trend with the increase of disintegration degree can be seen in Figure 4c, as the dotted line shows. However, there were two data point in the figure that departed far from the trend line. One of the two points was above the trend line, at 33.6% of disintegration degree, which was for the US disruption process; and another point was under the trend line, at 47.6% of disintegration degree, which was for the enzymatic process. Moreover, it was interesting that the “lipid yield” in US process was higher than that in enzymatic process. It might be explained that there were some differences in the disruption mechanisms of the two processes. In the enzymatic process, the lipids were not disturbed and separated with other substances, though cells were not yet integrated. However, in the US process, strong agitations were introduced into the cells, and not only the cells were broken, but also the lipids were well separated from other substances so that lipid extraction became easier.

### 2.5. Cell Morphology Observation

**Figure 5:**
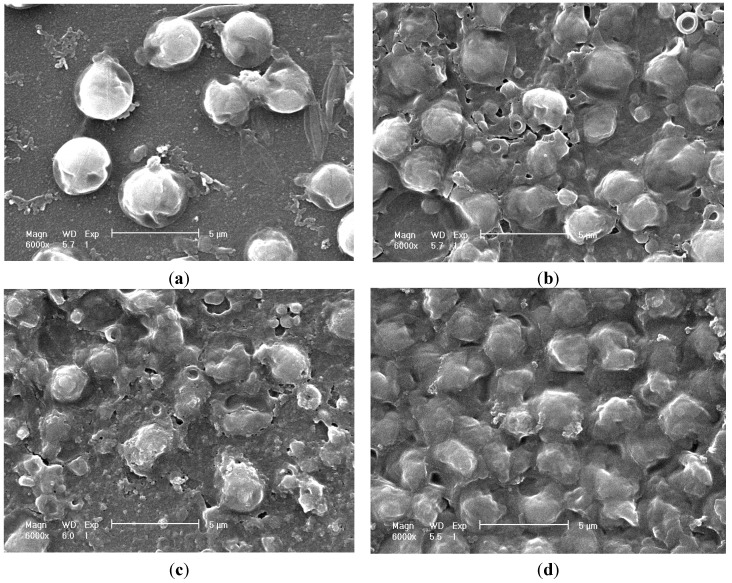
Scanning electron microscopy (SEM) images of algae cell before and after disruption (6000×). (**a**) SEM image of algae cells of *Neochloris oleoabundans*; (**b**) SEM image of cells disruption with ultrasonic wave; (**c**) SEM image of cells disruption with high-pressure homogenization; and (**d**) SEM image of cells disruption with enzymatic hydrolysis.

**Figure 6:**
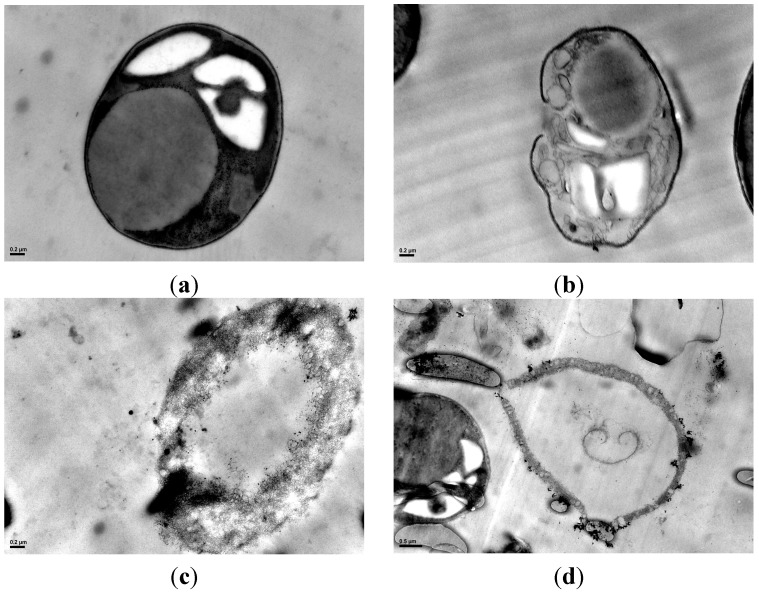
Transmission electron microscopy (TEM) images of algae cell before and after disruption. (**a**) TEM image of an intact cell of *Neochloris oleoabundans* (40,000×); (**b**) TEM image of an algal cell disrupted with ultrasonic wave (40,000×); (**c**) TEM image of an algal cell disrupted with high pressure homogenization (40,000×); and (**d**) TEM image of an algal cell disrupted with enzymatic hydrolysis (25,000×).

Figure 5 and Figure 6 show the images of the alga cells before and after disruptions. Data in Figure 5 was obtained through scanning electron microscopy (SEM), and in Figure 6 through transmission electron microscopy (TEM). A large lipid droplet in intact cell could be observed in Figure 6a (the area “L”), denoting the cell had high lipid content (the lipid content of the collected algal mud was measured to be 37% *w*/*w* of biomass via Soxhlet method as in Figure 4b). The cell fragment was obvious in the SEM images after high-pressure homogenization and ultrasonication. The cell was fragmented completely after homogenization, as shown in Figure 6c, and the cell treated by ultrasonication appeared disintegrated in Figure 6b. However, the cells treated by enzymatic hydrolysis appear to remain intact, as shown in Figure 5d. This situation can be explained as the enzymatic hydrolyzation partially decomposed the cell wall structure but did not disrupt the cells into pieces; as a result more cells could keep their intact morphology image. Though the enzymatically treated cells can remain intact for a certain period of time, they will be disintegrated after stirring, so the disruption degree after enzymatic hydrolysis can also be observed. However, enzymatic hydrolysis can substantially destroy the cell wall structure. As shown in Figure 6d, a cell wall had been broken and leakage of cell inclusion could be observed.

### 2.6. Discussion

In wet lipid extraction process, organic solvent has to first disperse into aqueous medium (if solvent volume is less than the medium like this research), and then dissolve free lipid outside cells and penetrates into cells to draw lipid out to medium. As the cells are wet, the intracellular system is aqueous. It is therefore much harder for solvent to access cells to dissolve intracellular lipid, compared with the dry process. Thus, it becomes critical for wet lipid extraction process that cells are disintegrated as completely as possible, so that the lipid can be released out of cell and become free lipid.

Analyzing disruption determination, there are several methods for disruption quantification, including cell counting, monitoring protein release, UV absorbance, turbidity, sample mass loss analysis, variations in viscosity and measuring the particle size distribution. A recent research [31] showed that all these quantification techniques present similar relationships between the measured extent of disruption and the disruption operation, though turbidity, particle sizing and UV absorbance generally gave more conservative estimates of the extent of cell disruption compared to protein release and cell counting. However, the disintegration measured by cell counting can be more precise for the fragment of cell. Therefore, in our current research, a cell counting method has been used for disruption quantification.

Ultrasonication does not usually give very high disruption efficiency and it costs much energy [25,29,38]. McMillan *et al*. [23] compared the cell disruptions of *Nannochloropsis oculata* by microwave, water bath, blender, ultrasonic and laser treatment, and the results showed that ultrasonication was least effective with a mean disruption of 67.66% ± 1.97% and an energy consumption of 132 MJ/L at a concentration of 1.8 × 10^8^ cell/mL. In our current experiment, ultrasonication was also not the best disruption method, as the cell disruption degree was only about half of that caused by high-pressure homogenization, and its energy consumption is 54 kJ/g dry algal mass (600 W 30 min, 1000 mL, mass concentration 20 g/L).

High-pressure homogenization also requires high energy in the reports [31,38]. In our research, a suspension of 1000 mL (mass concentration 20 g/L) was homogenized twice (4 min) by a homogenizer (4 kW) working at 80 MPa with a disruption degree of 83.07%, and the energy consumption is equivalent to 48 kJ/g of dry mass. This energy consumption is lower than the reported. The mass concentration of the suspension in our experiment is much higher (20 g/L) than that in the reported studies mentioned above, and this might be one of the factors reduced energy requirement as a calculation of per gram of mass. Comparing the ultrasonication result in this experiment, we note that with the similar levels of energy consumption, high-pressure homogenization can obtain higher cell disruption efficiency than ultrasonication.

Since cellulose is one of the dominant components of *N. oleoabundans* cell wall [35], cellulases should be effective to break cells. Moreover, proteases are also helpful to lyse the proteins embedded in the cell wall. For these reasons, a combination of cellulase and protease is necessary for a better lysis. As the cell wall skeleton is built from cellulose, the cellulase will take a more important role in the cell disruption compared to protease. Therefore, the combination pattern will be critical. If both cellulase and protease are put together, cellulase could be hydrolyzed by protease; if cellulase is put into the suspension first, it will avoid being attacked by protease, but will be hard to access the cellulose in the integral cell wall. In contrast, when protease is put into the suspension first it attacks proteins in the cell wall structure and makes it loosened, so the cellulase, added later, can access the substrate more easily, and higher lysis efficiency can be achieved. However, there are no detailed studies reported yet on the combination pattern of enzymes. In our present study, the cell disruption efficiency of different enzyme combinations has been compared, and with the best combination a three-fold lipid yield was obtained, compared with the undisrupted control sample.

Although enzymes can decompose the cell wall substances, complete fragment of cells may not be achieved until a large quantity of enzymes act for a long enough time, which also requires mechanical stirrings. Large quantity of enzymes also means high-energy consumption because enzyme production is also an energy-costing process. In the enzymatic-mechanical combination process, enzymatic treatment can significantly reduce the mechanical energy requirement. In our combination process, we co-apply a reduced quantity of enzymes comparing to the sole enzymatic process (papain: 2% to 1%; cellulase: 3% to 1%) with strength-decreased mechanical processes (high pressure homogenization: twice to once and 80 to 60 MPa; ultrasonication: 800 to 600 W), significantly increasing lipid yield. In our enzymatic-mechanically combined processes possessing the advantages of enzymatic lyses and mechanical disruption, the lipid yields were much higher compared to the sole process. Based on the lipid yield of Soxhlet method, the highest lipid recovery of enzyme-homogenization combined process was 92.6%.

The energy costs and lipid yields of sole processes and combined processes analysis are presented in Table 1, where the combination of enzymatic treatment and high-pressure homogenization demonstrates promise, producing a much higher lipid yield and lower disruption cost than any other process.

**Table 1:** Comparisons of costs and lipid yields in different disruption processes. 1. The value of un-extracted oil (lost in algal suspension) is calculated as 0.50 USD/kg, which is estimated according to the alga cultivation cost, and the oil loss is calculated by Soxhlet oil yield (7.2 g/L) minus the oil yield of selected process. 2. The prices of enzymes and electricity refer to the Chinese market. 3. The electricity cost in enzymatic process is neglected.

Disruption Processing	E	US	E + US	HPH	E + HPH
Oil recovery					
Oil yield (g/L)	2.35	3.84	5.55	4.96	6.67
Oil loss (g/L)	4.85	3.36	1.70	2.24	0.53
Count for oil loss (USD)	0.0024	0.0017	0.00065	0.0011	0.00027
Enzyme					
Papain (g/L)	0.4		0.2		0.2
Cellulase (g/L)	0.6		0.2		0.2
Cost for enzymes (USD)	0.0066		0.0023		0.0023
Electricity (for mechanical disruption)					
Electricity currency (A, 220 V)		3.5	2.5	13.5	10.5
Operation time (h)		0.5	0.5	0.07	0.035
Energy (kJ/L)		1386	990	712	277
Cost for electricity (USD)		0.0347	0.0243	0.0178	0.00693
Total disruption processing cost (USD)	0.009	0.0364	0.0273	0.0189	0.00953
Disruption cost to yielded oil (USD/kg)	3.83	9.81	5.41	3.81	1.43

## 3. Experimental Section

### 3.1. Strain and Culture Conditions

The strain *Neochloris oleoabundans* was purchased from UTEX the Culture Collection of Algae at the University of Texas in Austin (Austin, TX, USA). The alga was cultivated at 30 °C with continuous illumination of 300 μmoL·m^−2^·s^−1^ in a modified soil extract (SE) medium, composed of (per liter) 0.15 g K_2_HPO_4_·3H_2_O, 0.15 g MgSO_4_·7H_2_O, 0.05 g CaCl_2_·2H_2_O, 0.35 g KH_2_PO_4_, 0.05 g NaCl, 2.86 mg H_3_BO_3_, 1.81 mg MnCl_2_·4H_2_O, 0.22 mg ZnSO_4_·7H_2_O, 0.079 mg CuSO_4_·5H_2_O, 0.039 mg (NH_4_)_6_Mo_7_O_24_·4H_2_O, and 0.375 g NaNO_3_. The cultivation was carried out in five 3000 mL glass conical flasks. A CO_2_ enriched air stream (5% *v*/*v* CO_2_) was bubbled into the cultivation flasks from the bottom at a flow rate of 0.5 vvm (the ratio of air volume flowed per minute to the culture broth volume). The cultivation temperature was controlled by forced circulation of ambient air using fans and the ambient temperature in the cultivation room was controlled by an air conditioner. Agitation in the cultivation flasks was achieved by combined bubbling and magnetic stirrers. The cultivation was initiated at 0.04 g/L biomass concentration and performed over a period of seven days.

### 3.2. Measurements of Cell Density

Biomasses were determined routinely by measuring the optical density of samples at 600 nm (OD_600_). Biomass concentration was calculated by multiplying OD_600_ values with 0.4, a predetermined conversion factor converting the OD_600_ value to dry cell weight (DCW) from our primary research [2].

### 3.3. Biomass Harvest

At the end of batch cultivation, bobbling and agitation were stopped to precipitate for 4 h, and then, the supernatant of about half of the broth volume was dumped out and the subnatant was centrifuged at 4300× *g* for 10 min to collect microalgal mud, which was kept in 4 to 8 °C for no more than 12 h and used for further experiments.

### 3.4. Cell Disruption by Ultrasonic Wave

Microalgal mud was re-suspended with water to make algal cell suspensions at a concentration of 4 g/L. The suspensions were processed with an ultrasonicator (Model JY92-2DN, Nbscientz Biotechnology Co., Ltd.; Ningbo, China), which was equipped with a probe of 8 mm in diameter with the ability to deliver ultrasound at a frequency of 25 kHz with adjustable power up to 900 W. An algal cell suspension of 500 mL was treated with the ultrasonic probe immerged in the suspension and at a selected power (400, 600 or 800 W) for 50 min, and well mixing was contributed by a mechanical stirrer. The operation was conducted in a batch to maintain the temperature. The suspension was sampled for analysis before the ultrasonication and at every 10 min during ultrasonication.

### 3.5. Cell Disruption by Homogenization

Microalgal cell suspensions were prepared through re-suspending algal mud with water to make up the cell concentrations of 4, 8, 12, 16 and 20 g/L. Suspensions of 1000 mL at selected biomass concentrations were pumped through the valve of a high-pressure homogenizer (Model GJJ-0.03/100, flow rate 30 L/h, max. pressure 100 MPa, motor power 4 kW, Shanghai Nouni Light Industry Machinery Co., Ltd.; Shanghai, China) at a selected operating pressure of 40, 50, 60, 70 or 80 MPa. The suspensions were sampled (10 mL) for analysis before and after the homogenization.

### 3.6. Cell Disruption by Enzymatic Lyses

Suspensions of algal cells were prepared with the algal mud through dilution with water and the cell concentrations of the suspensions were adjusted to be 4 to 20 g/L. The prepared suspensions were treated by single enzyme of cellulase, papain or neutral protease (all enzymes were purchased from Imperial Jade Bio-technology Co., Ltd.; Yinchuan, China), the ratio of enzyme mass to algal biomass was from 1% to 6% (*w*/*w*), or by enzyme combinations of cellulase and protease as three combination patterns in Table 2.

**Table 2:** Enzymatic treatment combination patterns.

Patterns	Reaction Schedule
Pattern 1	(cellulase + papain), reacting for 3 h
Pattern 2	cellulase, reacting for 3 h → papain, reacting for 2 h
Pattern 3	papain, reacting for 2 h → cellulase, reacting for 3 h

After enzymatic treatment, the suspensions were stirred with a magnetic stirrer for 10 min, and then the disruption degrees of algal cells were measured immediately.

### 3.7. Cell Disruption by Combined Processes of Enzymatic Lyses and Mechanical Methods

An algal cell suspension at the biomass concentration of 20 g/L was treated firstly by 1% (*w*/*w*) papain for 2 h followed by 1% (*w*/*w*) cellulase for 3 h, and then was ultrasonicated at 600 W for 30 min or homogenized one pass at 80 MPa. Each suspension was sampled to determine the disruption degree and readily for lipid extraction.

### 3.8. Determination of Cell Disruption Degree

Microalgal cell suspensions were sampled for observation with a microscope. Samples were diluted with culture medium to a convenient concentration before observation. Intact cell count of each sample was determined with a standard Neubauer haemocytometer [38]. Only undamaged cells were counted as intact. The disruption degree was calculated as *D* = (*C*_0_ − *C*)/*C*_0_, where *C*_0_ and *C* were the intact cell count (cells/μL) of the sample before and after disruption, respectively.

### 3.9. Lipid Extraction

Algal cell suspensions of 1000 mL at 20 g/L of biomass were treated, respectively, by ultrasonication, homogenization, enzymes or their combinations at selected conditions, and then each suspension had 300 mL *n*-hexane added and was stirred for 4 h with a magnetic stirrer to extract lipid. After the extraction, the mixture was centrifuged to separate the water phase and organic solvent phase. Then, the hexane phase and the emulsified phase had water added and was further stirred to break emulsion and wash out the hydrophilious substances. The hexane phase was collected again through centrifugation and the lipid was obtained from the hexane phase by evaporating *n*-hexane. The extracted lipid was weighed after being dried in an oven at 80 °C for 2 h.

### 3.10. Morphological Observation

The samples of algal cells were prepared through being fixed with 2.5% (*v*/*v*) glutaraldehyde for 3 h, washed with 0.1 moL/L pH 7.4 phosphate buffer 3 times, fixed with 20 g/L osmic acid for 2 h and washed with phosphate buffer 3 times, and dehydrated successively with phosphate buffer containing 30%, 50%, 75% and 95% (*v*/*v*) ethanol for 3–5 min and with anhydrous ethanol for 5 min. The prepared samples finally were sprayed with gold for scanning electron microscopy or sliced for transmission electron microscopy observation.

### 3.11. Data Statistic Analysis

Each treatment was conducted with three replications, and the results were presented as mean ± SD (standard deviation). The data were subjected to analysis of variance using SPSS Version 12.0 (SPSS Inc., Chicago, IL, USA) software and differences (*p* < 0.05) between means were determined using *t*-test.

## 4. Conclusions

Though ultrasonication, high pressure homogenization and enzymatic treatment can all disrupt the microalgal cells efficiently, high-pressure homogenization can achieve most the efficient cell disruption of the three. In the enzymatic disruption process, cellulase in company with protease can provide higher disruption efficiency than either of the two enzymes solely, and the combination pattern of the enzymes is a critical factor in the enzymatic process. Avoiding being hydrolyzed by protease, cellulase should be provided to treat the algae cells after the protease is used. A combination of enzymatic treatment and mechanical technique can also obtain higher disruption efficiency than any sole process, and it has been proved that high-pressure homogenization combined with enzymes, can not only achieve more cell disruption and lipid recovery than any of the sole enzymatic or mechanical process, but also requires less energy consumption for processing, thus it possesses the lowest processing cost.
